# Estimating sparse functional brain networks with spatial constraints for MCI identification

**DOI:** 10.1371/journal.pone.0235039

**Published:** 2020-07-24

**Authors:** Yanfang Xue, Limei Zhang, Lishan Qiao, Dinggang Shen

**Affiliations:** 1 School of Mathematics, Liaocheng University, Liaocheng, China; 2 Department of Radiology and BRIC, University of North Carolina at Chapel Hill, Chapel Hill, North Carolina, United States of America; 3 Department of Brain and Cognitive Engineering, Korea University, Seoul, South Korea; Universidad Rey Juan Carlos, SPAIN

## Abstract

Functional brain network (FBN), estimated with functional magnetic resonance imaging (fMRI), has become a potentially useful way of diagnosing neurological disorders in their early stages by comparing the connectivity patterns between different brain regions across subjects. However, this depends, to a great extent, on the quality of the estimated FBNs, indicating that FBN estimation is a key step for the subsequent task of disorder identification. In the past decades, researchers have developed many methods to estimate FBNs, including Pearson’s correlation and (regularized) partial correlation, etc. Despite their widespread applications in current studies, most of the existing methods estimate FBNs only based on the dependency between the measured blood oxygen level dependent (BOLD) signals, which ignores spatial relationship of signals associated with different brain regions. Due to the space and material parsimony principle of our brain, we believe that the spatial distance between brain regions has an important influence on FBN topology. Therefore, in this paper, we assume that spatially neighboring brain regions tend to have stronger connections and/or share similar connections with others; based on this assumption, we propose two novel methods to estimate FBNs by incorporating the information of brain region distance into the estimation model. To validate the effectiveness of the proposed methods, we use the estimated FBNs to identify subjects with mild cognitive impairment (MCI) from normal controls (NCs). Experimental results show that the proposed methods are better than the baseline methods in the sense of MCI identification accuracy.

## Introduction

Alzheimer’s disease (AD) is an age-related, progressive neurodegenerative disease with the main characteristics of memory loss and cognitive decline. Currently, more than 35 million people suffer from AD all over the world [1], not only causing agony of losing memory for patients, but also bringing heavy financial burdens to the patient family and society. Unfortunately, researchers have not yet found an effective way of treating AD completely. Intervention is currently believed to play an important role in preventing or delaying AD at the stage of mild cognitive impairment (MCI).

To predict MCI (or AD as early as possible), researchers have explored many approaches from multiple aspects, including biochemistry [2], genetic [3], and brain imaging [4]. Especially, in recent years, functional magnetic resonance imaging (fMRI), which achieves blood oxygen level dependent (BOLD) signals, provides a noninvasive way of identifying subjects with MCI from normal controls (NCs). In practice, however, fMRI signals are arbitrarily scaled and have no unit [5], which causes the difficulty of comparing fMRI time series directly between different subjects. In contrast, fMRI-based functional brain network (FBN) has become a potentially effective tool to find informative patterns in fMRI data, and has been used to investigate MCI identification. In fact, with the help of FBN analysis, researchers recently have made a considerable progress in probing the mechanism of neurological diseases [6].

Normally, FBN is estimated by calculating the statistical dependency between the measured BOLD signals associated with different brain regions of interest (ROIs). The most classic method is Pearson’s correlation (PC), which is unfortunately sensitive to both direct and indirect relationship between ROIs and thus often results in false functional connections [5]. In contrast, partial correlation [7], as an alternative to PC, is only sensitive to direct relationship by regressing out the confounding effect from other ROIs. In fact, regardless of using which correlation-based method (without regularizers or postprocessing), the estimated FBN tends to be dense, when simply interpreting pairwise correlations between ROIs as weights of brain network connections. Therefore, a threshold or *L*_1_-regularizer is generally employed in correlation-based models for estimating sparse FBNs. The representative methods include regularized PC [8], sparse inverse covariance estimation [9], and sparse representation (SR) [10]. In addition, researchers have also developed many complex higher-order FBN estimation methods, as summarized in recent review papers [11, 12] for most of these methods.

Despite their continuous emergence, the current methods, to our best knowledge, only employ the data in fMRI signals to estimate FBNs, which ignores spatial relationship between these signals. In fact, due to the parsimony principle of our brain for saving the space and material, the strength of connection between two brain ROIs will decrease as their distance increases [13]. Here, we simply consider the friendship network as an analogy to FBN for explaining the meaning of spatial “constraints”. In particular, each person in the friendship network is a node (in analogy with ROI in FBN), while the social relations between two persons are defined as the edge (in analogy with the functional connection in FBN). If two persons are close in space, for example, studying in the same class or working in the same group, they 1) are likely to have a closer relationship, and/or 2) tend to share a more similar circle of friends. Therefore, we have reasons to assume that those spatially close ROIs may have a stronger functional connection, and/or share a more similar connection topology with other ROIs. Based on such an assumption, in this paper, we incorporate spatial distance information into the traditional model in two different ways, and *in turn* develop two novel FBN estimation methods. In order to verify the effectiveness of the proposed methods, we apply the estimated FBNs to identify subjects with MCI from NCs. The results show that our methods can achieve higher classification accuracy than the baseline methods.

The rest of this paper is organized as follows. In Section of *Related Works*, we review two representative FBN estimation methods. In Section of *Materials and Methods*, we introduce the source of the data and our proposed methods including our motivation, models and algorithms. In Section of *Experiments and Results*, we report the experimental setting and results. In Section of *Discussion*, we discuss our findings, and the strength and limitation of the proposed method. In the last section, we conclude the whole paper.

## Related work

As described earlier, researchers have developed many methods to estimate FBNs, including PC [14], partial correlation [7], regularized partial correlation [10], and some complex higher-order variants [12]. In this paper, we only focus on correlation-based methods, since they are empirically verified to be more sensitive than complex higher-order methods according to a recent comparative study [11].

### Pearson’s correlation

PC is the simplest correlation-based method to estimate FBNs. Suppose that the brain has been parcellated into *P* ROIs according to a certain atlas. Then, the functional connection *w*_*ij*_ between the *i*^*th*^ and *j*^*th*^ ROIs can be defined via PC can be computed as follows:

$$w_{ij}=\frac{\left({x_{i}-{\overset{-}{x}}_{i})}^{T}(x_{j}-{\overset{-}{x}}_{j}\right)}{\sqrt{{\left(x_{i}-{\overset{-}{x}}_{i}\right)}^{T}(x_{i}-{\overset{-}{x}}_{i})}\sqrt{{\left(x_{j}-{\overset{-}{x}}_{j}\right)}^{T}(x_{j}-{\overset{-}{x}}_{j})}}$$ (1)

wherewhere *x*_*i*_ ∈ *R*^*N*^ is the ${\overset{-}{x}}_{i}$ is the mean vector of the signal *x*_*i*_. Without loss of generality, we redefine $x_{i}=\frac{x_{i}-{\overset{-}{x}}_{i}}{\sqrt{{\left(x_{i}-{\overset{-}{x}}_{i}\right)}^{T}(x_{i}-{\overset{-}{x}}_{i})}}$. Then, the PC-based functional connectivity can be simplified as $w_{ij}={x_{i}}^{T}x_{j}$ that exactly corresponds to the optimal solution of the following problem:

$${min}_{w_{ij}}\sum_{i,j=1}^{P}{\left\|x_{i}-w_{ij}x_{j}\right\|}_{2}^{2}$$ (2)

where *P* is the number of ROIs.

### Sparse representation

As an alternative to PC, partial correlation is another commonly used FBN estimation method. Different from PC, partial correlation is only sensitive to indirect dependency between ROIs by regressing out the confounding effect from other ROIs. However, partial correlation needs to calculate the inverse of the sample covariance matrix explicitly or implicitly, causing an ill-posed problem. In order to obtain a stable estimation of FBN, an *L*_1_-regularizer is generally introduced into the partial correlation model, which results in many popular FBN estimation methods such as SR [15] given as follows:

$${min}_{w_{ij}}(\frac{1}{2}\sum_{i=1}^{P}{\left\|x_{i}-\sum_{j\neq i}w_{ij}x_{j}\right\|}_{2}^{2}+\lambda \sum_{j\neq i}\left|w_{ij}\right|)$$ (3)

Equivalently, Eq (3) can be simplified into the following matrix form:

$${min}_{W}(\frac{1}{2}{\left\|X-XW\right\|}_{F}^{2}+\lambda {\left\|W\right\|}_{1})$$ (4)

where *X* = [*x*_1_, *x*_2_, ⋯, *x*_*P*_] ∈ *R*^*N*×*P*^ is the fMRI data matrix, *W* = (*w*_*ij*_) ∈ *R*^*P*×*P*^ is the adjacency matrix of the estimated FBN, *λ* is a regularized parameter for controlling the balance of two terms in the objective function, ∥·∥_*F*_ and ∥·∥_1_ denote the *F*-norm and *L*_1_-norm of a matrix, respectively.

## Materials and methods

### Data acquisition and preprocessing

In this paper, we use the dataset that were collected in Geneva University Hospital. It shares the same data source as previous works [16–18]. The raw dataset includes 60 subjects with MCI and 350 NCs. However, in order to avoid the class imbalance problem, the same number of NCs as the subjects with MCI was randomly selected from the dataset. After data preprocessing, 45 MCIs and 46NCs were left in our experiment, and the demographic information of these subjects is shown in Table 1. All the preprocessed data can be downloaded from https://github.com/X-Yanfang/xyf-DATA. The original fMRI signals of the subjects were obtained by 3.0T Philips MR scanner, and the imaging parameters are set as follows: the obtained matrix size is 74 × 74 with 45 slices, voxel size is 2.97 × 2.97 × 3 *mm*^3^, TE is 30ms, and TR is 3000ms with 180 repetition. The 3-dimensional coordinates of voxels are extracted from the automated anatomical labeling (AAL) template [19]. All procedures performed in this study were in accordance with the ethical standards of the institutional and/or national research committee and with the 1964 Helsinki declaration and its later amendments or comparable ethical standards, and this study is supported by the ethics committee of Geneva University Hospital [16].

**Table 1:** 

	MCI	NC
Number of subjects (male/female)	25/20	14/32
Age (mean ± SD)	74.13 ± 6.68	73.5 ± 3.50
MMSE (mean ± SD)	27.71 ± 1.73	28.10 ± 1.35

Noises caused by, for example, the scanner and head motion, have large influence on the FBN estimation and analysis. In order to reduce the influence, a preprocessing pipeline is used in this paper to improve fMRI data quality prior to FBN estimation. In particular, for each subject the first ten volumes in the fMRI time course are removed for signal stabilization. Then, the remaining volumes were processed via Statistical Parametric Mapping (http://www.fil.ion.ucl.ac.uk/spm/) and DPABI [20] according to a popularly-used scheme. The main steps include 1) correcting slice timing and head motion; 2) regressing out nuisance signals with Friston 24-parameters of head motion; 3) registering the corrected images to Montreal Neurological Institute standard space; 4) spatially smoothing the signals with the full-width-half-maximum of 4mm, and filtering the signals using a band pass frequencies between 0.01 and 0.1. After that, we parcel the brain according to AAL atlas, and extract the mean signal from each ROI. Note that we use AAL atlas mainly due to its popularity. In the discussion section, we will give more details of the atlas selection and its possible effect on the results. As a result, we get a data matrix *X* ∈ *R*^*N*×*P*^ (with *N* = 91 as the number of ROIs, and *P* = 80 as the length of time series) that is the main material for estimating FBNs. Please see (16, 18) for more details of the data preprocessing.

### Proposed methods

As discussed in the introduction section, our brain, despite its extreme complexity, is believed to organize similar to many existing physical system/networks in which the spatially close elements tend to have a stronger relationship. In fact, recent researches have also shown that spatially proximal ROIs have a higher possibility of connection than spatially distant ROIs due to the parsimony principle of wiring cost [13].

**Fig 1:**
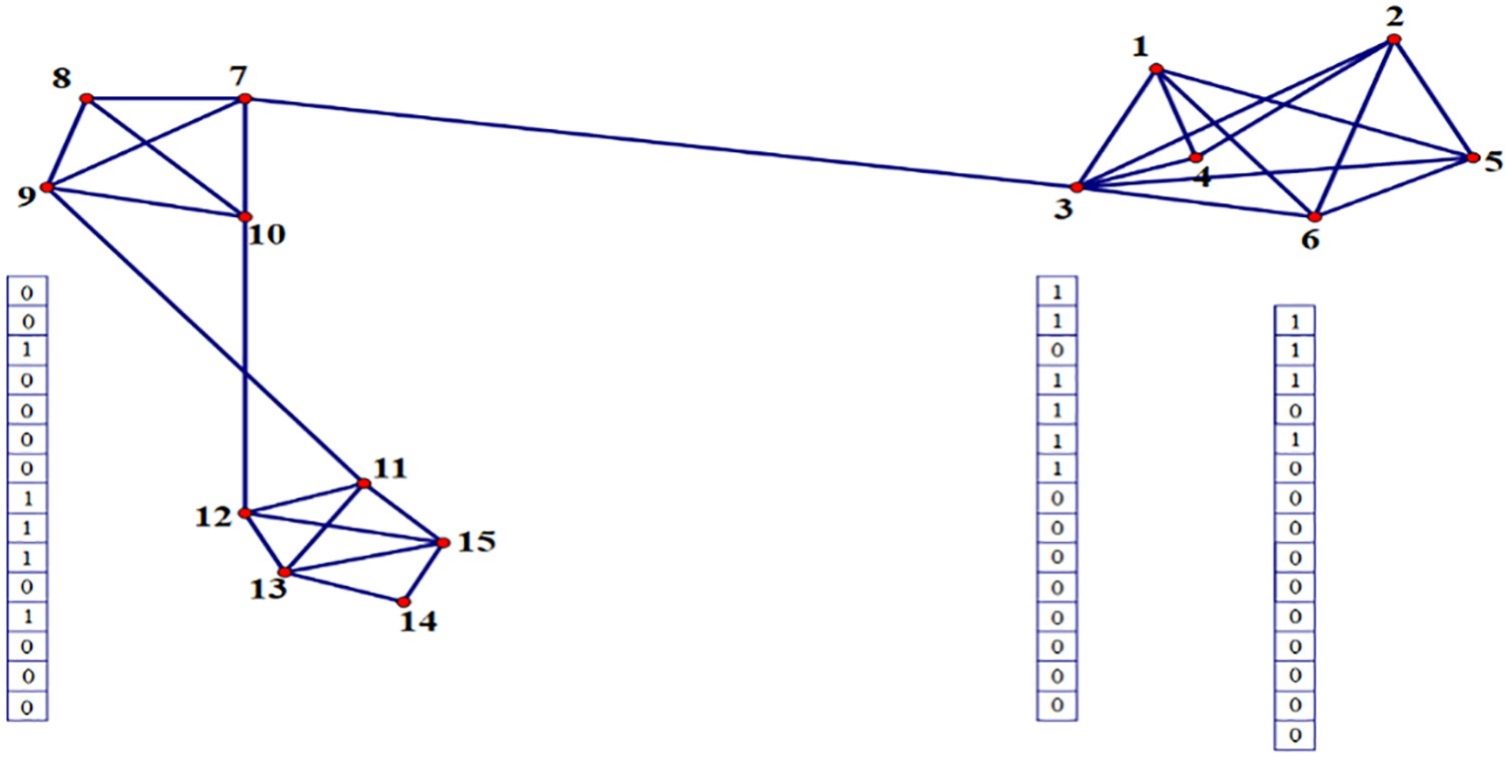
An illustrative example for explaining the motivation or assumption behind the proposed methods. The binary column vector in the figure denotes the connection pattern between the current node and other nodes. If two nodes (e.g., 3 and 6) are spatially closed, we assume they have a direct connection with a higher probability and share a similar connection pattern with other nodes.

In Fig 1, we provide a simple toy network for explaining the above viewpoint intuitively, as well as illustrating our motivation or assumption used in the newly proposed FBN estimation methods that will be discussed shortly. First, as shown in Fig 1, there are edges between some spatially close nodes (e.g., nodes 3 and 6), while usually no edge between spatially distant nodes (e.g., nodes 3 and 9). Regarding the relationship between the connection strength and spatial distance, Kaiser and Hilgetag investigated the fiber length distribution of cortical/nervous connections in the macaque and C. elegans, and found that most connections tend to be short in space [21]. Similar trend has been noted in human FBNs estimated with fMRI data [22]. In particular, several studies revealed that the strength of functional connections decreases with the spatial distance following a power-law [22–25]. Therefore, a natural assumption (Assumption I) is that *the connection strength in an FBN is inversely proportional to the distance of different RO*Is.

Further, more complex interaction between ROIs has been characterized by a higher-order pattern (e.g., the correlations among different edges, or correlation’s correlation) in several recent studies [26, 27]. This inspires us to give Assumption II that *the spatial distance also affects the network connections in a higher-order way*. In other words, *spatially close nodes share the similar connection topology with other nodes*. For example, the spatially close nodes 3 and 6 have a similar connection pattern with other nodes, as indicated by the binary vector in Fig 1. In contrast, the connection pattern associated with the remote node 9 is different.

Now, based on the above assumptions, we develop two novel FBN estimation methods by incorporating the spatial distance information into the traditional SR model in both low- and high-order ways, respectively.

#### Method 1: Estimating FBN based on assumption I

Based on Assumption I, we propose to estimate FBN by the following optimization problem:

$${min}_{w_{ij}}(\frac{1}{2}\sum_{i=1}^{P}{\left\|x_{i}-\sum_{j\neq i}w_{ij}x_{j}\right\|}_{2}^{2}+\lambda \sum_{j\neq i}c_{ij}|w_{ij}|)$$ (5)

In Eq (5), we use the same data-fitting term as SR for capturing the partial correlation structure. The only difference is that a weight *c*_*ij*_ is introduced into the regularized term for strengthening/weakening the functional connectivity according to the spatial distance between different ROIs. In our method, we define *c*_*ij*_ = *d*_*ij*_/*d*_*max*_, where *d*_*ij*_ is the spatial distance between the *i*^*th*^ and *j*^*th*^ ROIs, and *d*_*max*_ is the maximum value of *d*_*ij*_ for *i*, *j* = 1, 2, ⋯, *P*. More specifically, the spatial distance *d*_*ij*_ is defined as follows,

$$d_{ij}=\frac{1}{n_{i}n_{j}}\sum_{a=1}^{n_{i}}\sum_{b=1}^{n_{j}}{\left\|R_{i_{a}}-R_{j_{b}}\right\|}_{2}$$ (6)

where $R_{i_{a}}$ denotes the 3-dimensional coordinates of the *a*^*th*^ voxels in the *i*^*th*^ ROI, *n*_*i*_ and *n*_*j*_ are the numbers of voxels in the *i*^*th*^ and *j*^*th*^ ROIs, respectively. Mathematically, Eq (5) can be simplified into the following matrix form:

$${min}_{W}(\frac{1}{2}{\left\|X-XW\right\|}_{F}^{2}+\lambda {\left\|C\circ W\right\|}_{1})$$ (7)

where *C* = (*c*_*ij*_) ∈ *R*^*P*×*P*^ is the coefficient matrix whose elements are the normalized distance between ROIs, and ∘ denotes Hadamard product of two matrices.

Note that the objective function of Eq (7) is convex, but non-differentiable due to the weighted *L*_1_-regularizer. Therefore, in this paper we use the proximal method [28] to solve Eq (7). In particular, we first calculate the gradient of the data fitting term denoted by $f(W)=\frac{1}{2}{\left\|X-XW\right\|}_{F}^{2}$, and get ∇_*W*_*f*(*W*) = *X*^*T*^(*XW* − *X*). As a result, we have the following gradient descent step:

$$W_{k+1}=W_{k}-\alpha_{k}\nabla f(W_{k})$$ (8)

where *α*_*k*_ is the step size of the *k*^*th*^ iteration. Then, we use the proximal operation for *λ*∥*C* ∘ *W*∥_1_, defined as follows:

$${proximal}_{\lambda {\left\|C\circ W\right\|}_{1}}=sign(w_{ij})*max\left\{|w_{ij}|-\lambda \times c_{ij},0\right\}$$ (9)

to map the current *W* into a feasible region. As a result, we get a simple algorithm for solving Eq (7), as shown in Table 2.

**Table 2:** Algorithm for solving Eq (7).

Input: *X*, *C*, *λ*
Iterate
1. *W* ← *W* − *α*_*k*_(*X*^*T*^(*XW* − *X*))
2. $W\leftarrow {proximal}_{\lambda {\left\|C\circ W\right\|}_{1}}=sign(w_{ij})*max\left\{|w_{ij}|-\lambda \times c_{ij},0\right\}$
End
Output: *W*

#### Method 2: Estimating FBN based on assumption II

According to our previous discussion, if two ROIs are close in space, they are more likely to share a similar connection topology/pattern. Therefore, based on Assumption II we propose the second FBN estimation model as follows:

$${min}_{w_{ij}}(\frac{1}{2}\sum_{i=1}^{P}{\left\|x_{i}-\sum_{j\neq i}w_{ij}x_{j}\right\|}_{2}^{2}+\frac{\lambda_{1}}{2}\sum_{i,j=1}^{P}{\left\|w_{i}-w_{j}\right\|}_{2}^{2}s_{ij}+\lambda_{2}\sum_{j\neq i}\left|w_{ij}\right|)$$ (10)

where *w*_*i*_ is the *i*^*th*^ column of the network adjacency matrix *W*, indicating the connection pattern of the *i*^*th*^ node with other nodes. *λ*_1_ and *λ*_2_ are the control parameters used to balance the tradeoff among the three terms in the objective function.

Compared with the traditional SR reviewed in Eq (3), a new regularizer, i.e., $\sum_{i,j=1}^{P}{\left\|w_{i}-w_{j}\right\|}_{2}^{2}s_{ij}$, is introduced into the proposed model for constraining the spatially close ROIs to have more similar connection patterns. In particular, *s*_*ij*_ is inversely proportional to the spatial distance, reflecting the similarity of the connection topology between two ROIs, and is defined as follows:

$$s_{ij}=e^{-d_{ij}}$$ (11)

where *d*_*ij*_ is given in Eq (6).

With a series of mathematical formulation, Eq (10) can be simplified into the following matrix form:

$${min}_{W}(\frac{1}{2}{\left\|X-XW\right\|}_{F}^{2}+\frac{\lambda_{1}}{2}tr(W(D-S)W^{T})+\lambda_{2}{\left\|W\right\|}_{1})$$ (12)

where *S* = (*s*_*ij*_) ∈ *R*^*P*×*P*^ is the defined similarity matrix in Eq (11), *D* is a diagonal matrix whose diagonal element is the row sum of *S*, i.e., $D_{jj}=\sum_{i=1}^{P}s_{ij},\;j=1,2,\cdots ,P$. It can be noted that *D* − *S* is in fact the graph Laplacian of *S*.

Similar to Method 1, the proximal method is used to solve Eq (12). Specifically, we consider the first two terms in Eq (12) as a whole, and denote it as $f(W)=\frac{1}{2}{\left\|X-XW\right\|}_{F}^{2}+\frac{\lambda_{1}}{2}tr(W(D-S)W^{T})$. Then, we can easily get its derivative ∇_*W*_*f*(*W*) = *X*^*T*^(*XW* − *X*) + *λ*_1_*W*(*D* − *S*). As a result, we have the following algorithm to solve Eq (12), as shown in Table 3.

**Table 3:** Algorithm for solving Eq (12).

Input: *X*, *S*, *λ*_1_, *λ*_2_
Iterate
1. *W* ← *W* − *α*_*k*_(*X*^*T*^(*XW* − *X*) + *λ*_1_*W*(*D* − *S*))
2. $W\leftarrow {\text{proximal}}_{\lambda_{2}{\left\|W\right\|}_{1}}=\text{sign}(w_{ij})*\text{max}\{|w_{ij}|-\lambda_{2},0\}$
End
Output: *W*

## Experiments and result

### Feature selection and classification based on estimated FBNs

**Fig 2:**
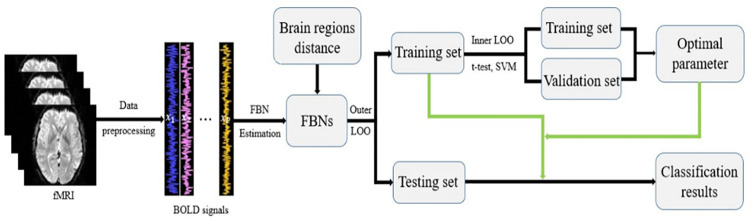
The experimental pipeline.

After obtaining the “clean” fMRI data, we estimate FBNs based on different methods, and compare their performance by a systematical experiment consisting of several main steps as shown in Fig 2.

**Step 1**: Estimating FBNs based on different methods and parametric values. For PC, we estimate FBNs by removing a percentage of weak connections, where the candidate percentage is selected in the set of [100%, 90%, ⋯, 10%]. For SR and Method 1, we use different values of the regularization parameter in the range of [0.001, 0.002, ⋯, 0.009, 0.01]. For Method 2, the two regularized parameters, *λ*_1_ and *λ*_2_, are selected in the ranges of [2^−10^, 2^−9^, ⋯, 2^−2^, 2^−1^] and [0.001, 0.002, ⋯, 0.009, 0.01], respectively.
**Step 2**: Feature selection and classification. We use t-test (with *p*-value equal to 0.01) for selecting discriminative features, and use the linear support vector machines (SVM) (with default parameter *C* = 1) for performing classification tasks. Due to the limitation of sample size, we use the leave-one-out (LOO) cross validation to calculate the performance of different methods. Specifically, for the 91 subjects in the dataset, 90 of them are used for training, while the remaining one is used for testing. After a complete loop, we will obtain 91 classifiers, and the final classification accuracy is defined as:
  $$accuracy=\frac{the\;number\;of\;correct\;classification}{the\;number\;of\;trained\;classifiers},$$ (13)

Since the parameters involved in the FBN estimation models may affect the structure of brain network, we select the optimal parametric values by an inner LOO cross validation on the training set, as shown in Fig 2. In light of this, we will obtain a series of classification results in the inner LOO. Lastly, we determine the parametric values corresponding to the best classification accuracy, and use the FBNs estimated by the optimal parametric value to conduct the outer LOO for achieving the final classification results.

### Experimental results

A set of quantitative measurements, including accuracy, sensitivity and specificity, are used to evaluate the classification performance. Their definitions are given as follows:

$$Accuracy=\frac{TP+TN}{TP+FP+TN+FN}$$ (14)

$$Sensitivity=\frac{TP}{TP+FN}$$ (15)

$$specificity=\frac{TN}{TN+FP}$$ (16)

where *TP* is the number of positive subjects correctly classified in the MCI identification task, and *FN* is the number of negative subjects incorrectly classified in the MCI identification task. Accordingly, *TN* and *FP* are the numbers of their corresponding subjects, respectively. As a result, the classification performances are reported in Table 4.

**Table 4:** The classification performance of Method 2.

	Accuracy	Sensitivity	specificity
PC	60.44%	57.78%	63.04%
SR	67.03%	62.22%	71.74%
Method 1	71.43%	75.56%	67.39%
Method 2	76.92%	82.22%	71.74%

As shown in Table 4, we can find that the proposed methods are superior to the baseline methods. Hence, we argue that the spatial distance between different brain regions may play a potentially important role in estimating FBNs. Furthermore, we note that Method 2 works better than Method 1. In our opinion, this result is possibly caused by the fact that Method 1 is based on a too harsh assumption that two brain regions have stronger functional connection if they are close in spatial location, while Method 2 is based on a relatively moderate assumption that considers the distance information in a higher-order way.

**Fig 3:**
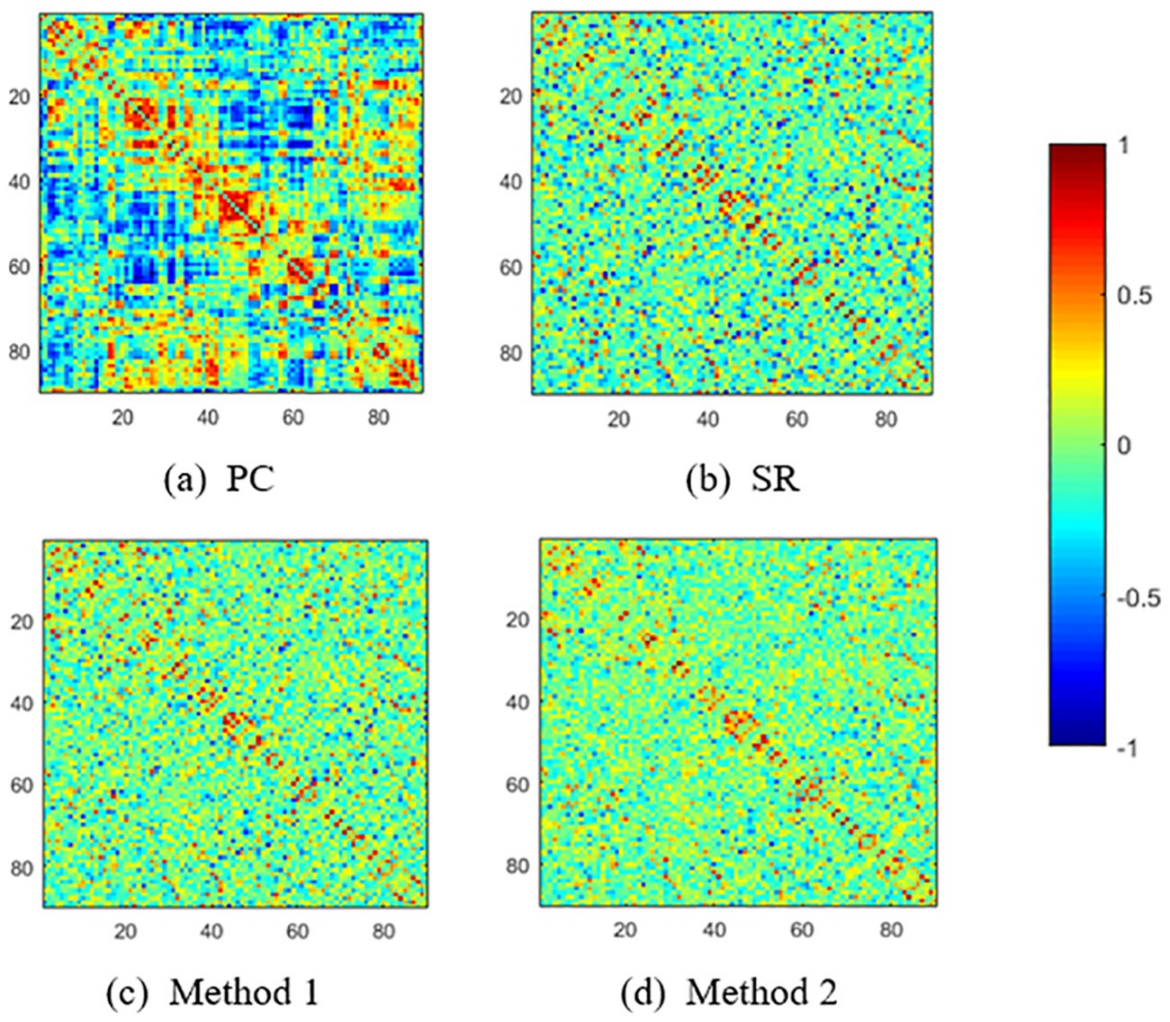
FBNs estimated by 4 different methods.

For an intuitive comparison, we visualize the FBNs estimated by PC, SR and our proposed methods, respectively, in Fig 3. It can be seen that the PC-based FBN is dense, since the pairwise full correlation is used to model the network adjacency matrix. As a result, it will lead to more false connections, and in turn affect the final classification accuracy. In contrast, SR and our methods can obtain spare FBNs by removing some weak or potentially noisy connections from the estimated FBN due to the *L*_1_ regularizer. Note that, the sparsity plays an important role in FBN estimation, but over-sparsity will cause the loss of useful discriminative connections. Therefore, we determine the suitable sparsity when estimating FBNs by a nested cross validation on the training data. Particularly, compared with the traditional SR and Method 1, the proposed Method 2 tends to achieve a cleaner FBN with clear modularity structures, as shown in Fig 3(d). Note that the density of networks estimated with the proposed methods may vary from subject to subject, due to the fact that the L_1-regularizer controls the network density indirectly, and the nested LOO CV automatically assigns an optimal value for the regularized parameter.

### Sensitivity to parameters

**Fig 4:**
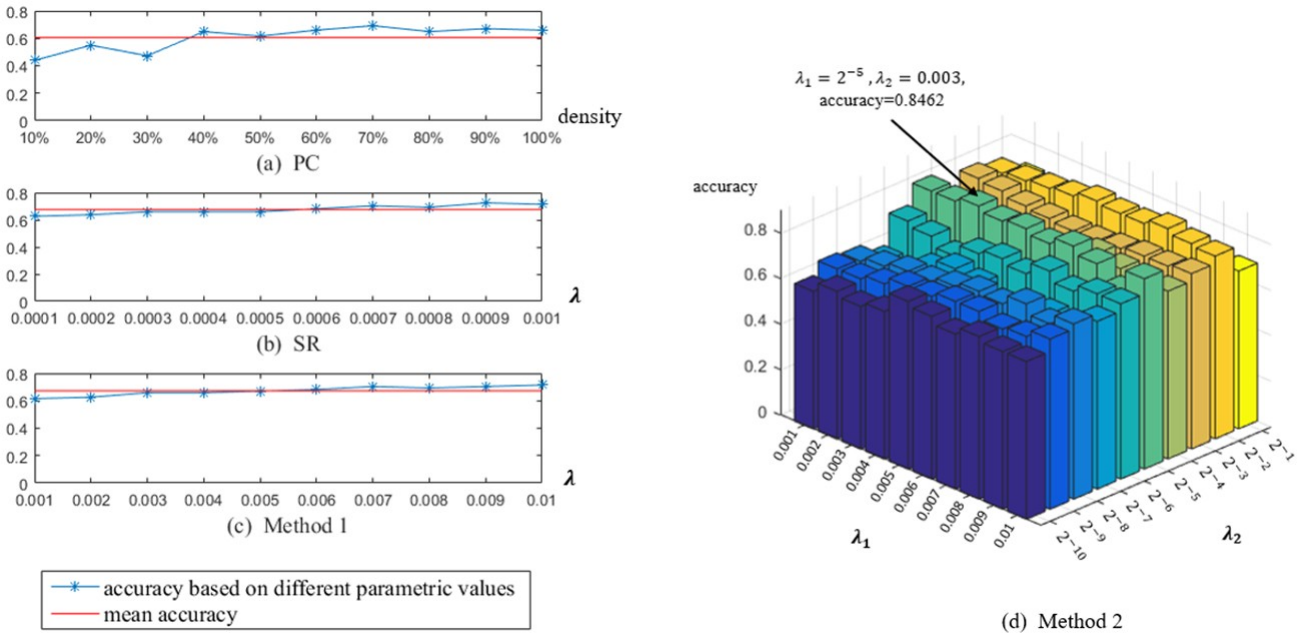
Classification accuracy by PC, SR and the proposed methods based on different parametric values. The horizontal axis represents network density (a) or regularization parameter (b-d), and the vertical axis represents the classification accuracy.

Parameters generally have an important influence on the FBN structure and the subsequent classification accuracy. In order to investigate the sensitivity of different methods to the involved parameters, we compute the classification accuracy under different parametric combinations, and report the results in Fig 4. Since there are two parameters in Method 2, we show the classification accuracy in a 2-dimensional bar graph. It can be observed from Fig 4 that the classification accuracy fluctuates heavily with the change of the parametric values, indicating that most of the FBN estimation methods are sensitive to the free parameters.

As shown in Fig 4, the classification accuracies fluctuate with the change of the parametric values. In particular, for PC-based FBNs, the optimal parametric values (i.e., network density) is 70%, and we note that an over-sparsity instead results in a drop of classification accuracy. For SR and Method 1, the *L*_1_-regularizer is introduced into the FBN estimation model for removing the weak connections and controlling the density of the estimated FBNs. From Fig 4(b) and 4(c), we observe that the optimal values of parameter λ are 0.009 and 0.01 for SR and Method 1, respectively. Note that two regularized parameters are involved in Method 2. One (*λ*_2_) is used for controlling the density of the FBNs, while the other (*λ*_1_) is used for modelling the similarity of two ROIs. As shown in Fig 4(d), *λ*_1_ = 2^−5^ and *λ*_2_ = 0.003 is the optimal combination for the two parameters. Therefore, for our proposed methods, we suggest setting the parametric value for *L*_1_-regularizer within the range of [10^−3^, 10^−2^], while the parametric value for high-order regularizer (in Method 2) within the range of [2^−5^, 2^−3^].

### Influence of parameter *c*_*ij*_ on results

Parameter *c*_*ij*_, as the function of the *d*_*ij*_, is used for strengthening/weakening the functional connectivity. We expect that the larger distance suppresses the connection, while the closer distance promotes the connection. In light of this, there are many definitions for *c*_*ij*_. To investigate their effects, we compare the classification results by defining different *c*_*ij*_, and report the results in Table 5. As shown in Table 5, the classification results are slightly affected by different definitions of *c*_*ij*_.

**Table 5:** The classification results of Method 1 under different value of *c*_*ij*_.

	Accuracy	Sensitivity	specificity
cij=dijdmax	71.43%	75.56%	67.39%
cij=dij2dmax	71.43%	73.33%	69.57%
cij=dij3dmax	70.33%	71.11%	69.57%

### Discriminative features

**Fig 5:**
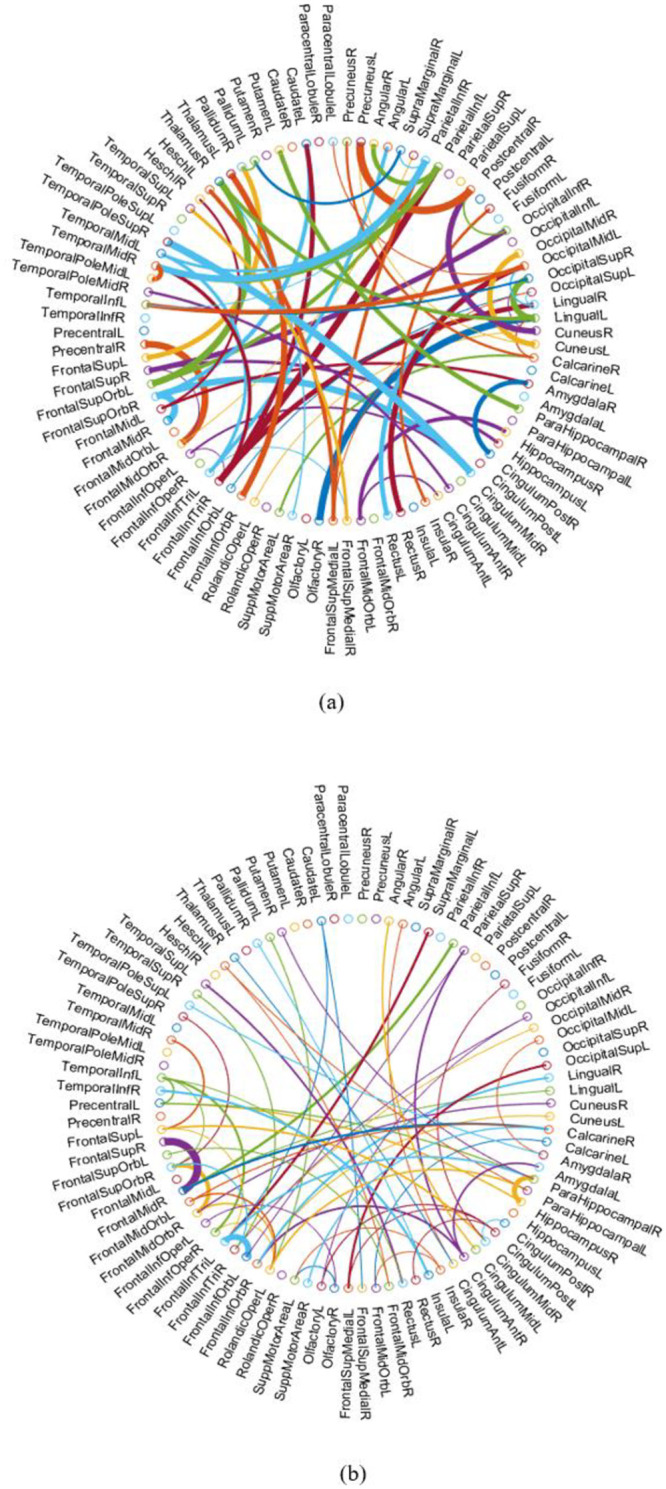
The selected features/connections based on t-test (a) and the weighs of SVM classifier (b). The color is set randomly for better visualization, and the width of the arc represents the discriminability of the corresponding connection.

In addition to the classification accuracy itself, a more informative aspect is which features/connections in FBN contribute to the final accuracy. In this paper, we find the discriminative connections in two different ways. For the first way, we record the top features selected by t-test with the default p-value of 0.01 in each loop of the cross validation, and report the common features in Fig 5(a). For the second way, we select discriminative features according to the mean of the absolute weights derived from SVM classifier in all loops of cross validation. It is generally believed that the larger the absolute weight of the corresponding feature is, the more discriminative this feature could be. Accordingly, the result is shown in Fig 5(b). Finally, we consider the features selected simultaneously by the two ways contributing the most to the MCI identification. Specially, the brain regions involved in the selected features by both ways include precuneus, amygdala, para-hippocampal. This finding is consistent with several previous studies on MCI [29, 30].

### FBN estimation with simulated data

**Fig 6:**
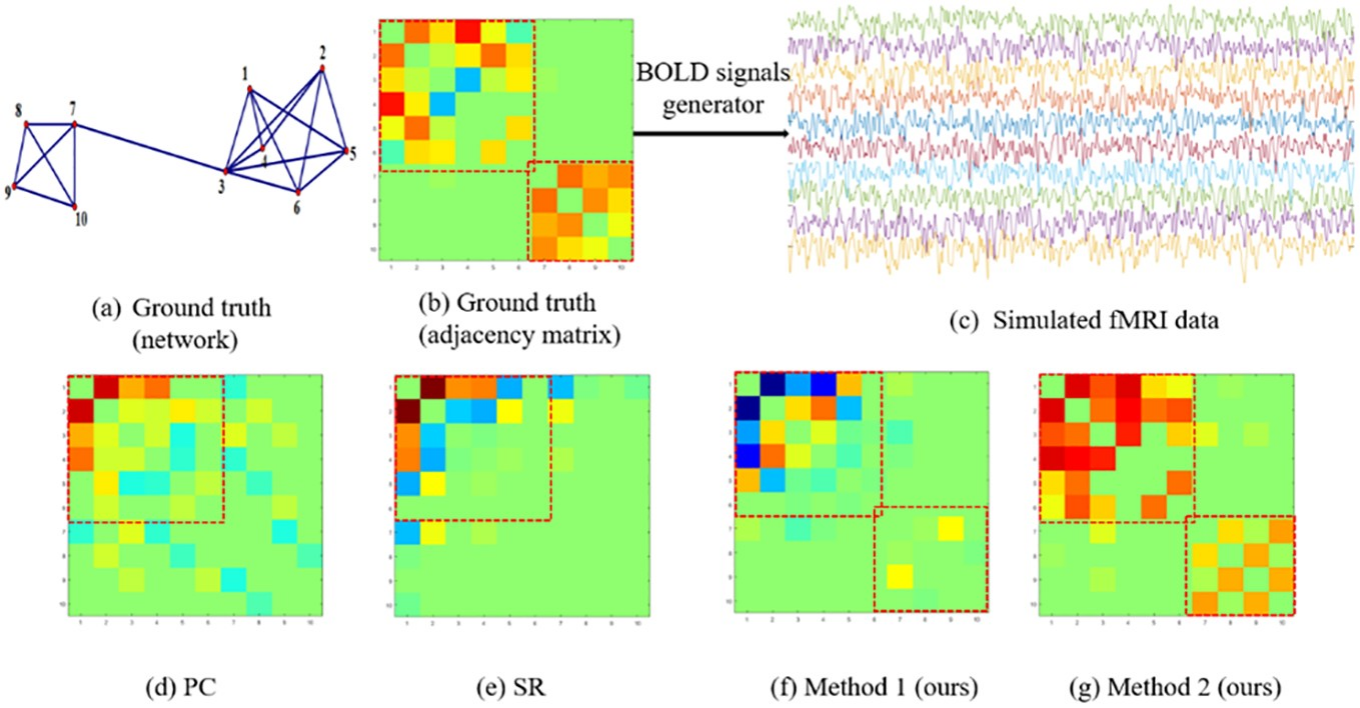
(a-b) The given network as ground truth; (c) The simulated BOLD signals generated by MULAN toolbox (https://github.com/HuifangWang/MULAN); (d)-(g) The estimated FBNs by running four different methods based on the simulated BOLD signals in (c).

Besides experiments on the real data set, we also estimate FBNs based on a set of simulated BOLD signals for evaluating the generalizability of the proposed methods and analyzing their ability to detect the network structures. First, we extract a subnetwork with 10 nodes from Fig 1 as the ground truth. As shown in Fig 6(a)–6(b), the nodes in the given network can be parcellated into two groups, indicating that the ground truth of FBN has a clear modularity structure. Then, based on the ground-truth network, we generate simulated BOLD signals with 1500 time points using MULAN toolbox [31]. Note that all the parametric values used in MULAN generator are provided by default in the supplementary material of [31]. In Fig 6(c), we show the 10 simulated BOLD signal series associated with 10 nodes. Finally, we estimate FBNs based on the simulated BOLD signals using different methods, and visualize the estimated results in Fig 6(d)–6(g).

From Fig 6, we have the following observations: 1) PC can only detect one of the network modules, and lead to some false connections. 2) SR can remove the weak (potentially false) connections, which makes the adjacency matrix look cleaner. However, it cannot recover the original network structure as PC. 3) The proposed Method 1 can roughly detect the network structure, but, comparing to the ground truth, there are some false connections between (and within) the two modules. 4) Different from the other three methods, our Method 2 can *not only* detect the original network structure, *but also* include the fewest false connections. Further, we also quantify the similarity between the estimated brain networks and ground truth by Pearson’s correlation coefficient as shown in Table 6. The results show that Method 2 achieves the highest similarity to the ground truth. This is consistent with the above observations in Fig 6(d)–6(g).

**Table 6:** The similarity between ground truth network and the generated FBNs.

	PC	SR	Method 1	Method 2
Ground truth	43.99%	50.13%	51.02%	70.04%

## Discussion

In this work, we propose two SR-based methods by combining the spatial information into the FBN estimation model in both low-order (Method 1) and high-order (Method 2) ways. Especially for Method 2, the experimental results show that its performance is better than other methods, including PC, SR and Method 1. This may be due to the fact that we do not only take the distance between different ROIs into account, but also attempt to model higher correlation (e.g., the correlation among different edges [30] or correlations’ correlation [24]) in the model. In addition, we note that there are more free parameters (*λ*_1_ & *λ*_2_) in Method 2 than other methods, which provides more flexibility to the model. However, the fact of more free parameters is not necessarily the reason for improving the performance that largely depends on whether the optimal parameter can be found in the training dataset. In our experiment, the optimal parametric value is selected by an inner CV on the training data, and Method 2 (under the selected optimal parametric value) is better than other methods. Although empirical results show the effectiveness of our proposed methods, there are many aspects that need to be further explored in the future. Therefore, in what follows, we give a brief discussion on several issues that the readers might be interested in.

For defining ROIs, researchers have developed many schemes, which can be roughly separated into atlas-based and data-driven methods. Atlas-based methods, such as AAL [19], anatomical Harvard-Oxford (HO) [32], Automatic Non-linear Imaging Matching and Anatomical Labeling (ANIMAL) [33], and Bootstrap Analysis of Stable Clusters (BASC) atlas [34], expect that the voxels within the same ROI tend to share the similar structure or function. In practice, however, this kind of methods generally suffer from low consistency [33, 35], partially due to individual difference, boundary vagueness and data unreliability. Additionally, the final BOLD signal is extracted by averaging on the voxels within a ROI, which results in information loss, and *in turn* makes the estimated FBNs include some unreliable connections [36].

As an alternative to atlas-based methods, data-driven methods directly work on the used dataset, and thus can relieve the low consistency issue to some extent. The popular methods for learning ROIs from data include clustering methods [37] and group independent component analysis methods (GICA) [38]. The clustering methods separate voxels into functionally homogeneous parcels, and define each parcel as a ROI. In contrast, GICA methods decompose the data into some components, each of which corresponds to a spatial map as the ROI. However, for data-driven methods, selecting the number of clusters or components is an exceedingly challenging problem. This is one of reasons why we simply use AAL atlas for parceling the brain in this paper. Another reason we use AAL is its popularity. For example, in a recent study [39], Brown et al. reviewed 77 works, in which AAL is the most commonly used method to define ROIs. In light of this, it is convenient for researchers to compare their findings with other studies. In summary, there is no consensus as to which ROI definition scheme is the best [5]. A recent study [40] systematically evaluated the influences of different aspects (including atlas selection, FBN estimation and classification design) on the FBN-based classification accuracy, and shown that, relative to the two others, the selection of atlas brings smaller influence on the final results.

For the performance improvement of the proposed methods in terms of classification accuracy, more information (e.g., spatial distance between ROIs) is included in this paper for estimating FBNs. For the spatial distance information, it can be uniquely (at least easily) determined in the voxel scale. However, in the ROI level, determining the spatial distance is not a trivial problem, because it heavily depends on both the calculation methods and the definition of ROIs. In [41], Alexander-Bloch et al. calculate spatial information using Euclidean distance between different ROIs centroids. In this paper, we define the distance as the average of the distances between all the voxel pairs in different ROIs. However, it is difficult for these methods to accurately assign physical distance between the ROIs with different sizes. For example, the distance between the two physically adjacent ROIs with big sizes may be greater than the distance between two nonadjacent ROIs with small sizes. Therefore, application of the distance between ROIs should be cautious, and further study is needed for designing a “good” metric to measuring distance between the ROIs with different shapes and sizes.

## Conclusion

PC and SR are the two baseline methods to estimate FBNs. PC, as the simplest method, is sensitive to indirect relationship between BOLD signals. In contrast, SR *not only* regresses out the indirect confounding effect before calculating the correlation between ROIs, *but also* incorporates the sparse prior into the FBN estimation model. However, SR estimates FBN only based on BOLD signals, yet ignoring their spatial location. Therefore, in this paper we propose two novel methods to estimate FBN by incorporating spatial distance information into the estimation model in two different ways. In order to verify the effectiveness of proposed methods, we conduct experiments based on the estimated FBNs. The results show that the proposed methods obtain a superior performance in the task of MCI classification. Therefore, we argue that the distance between different brain regions may play an important role in improving the quality of the estimated FBNs. In the future, we plan to conduct more experiments to evaluate the influence of the spatial distance on other FBN estimation methods.
