# Correction: Estimating sparse functional brain networks with spatial constraints for MCI identification

**DOI:** 10.1371/journal.pone.0253995

**Published:** 2021-06-24

**Authors:** Yanfang Xue, Limei Zhang, Lishan Qiao, Dinggang Shen

Affiliation 2 for author Dinggang Shen is incorrect. The correct affiliation 2 is: Department of Research and Development, Shanghai United Imaging Intelligence Co., Ltd., Shanghai, China.
